# SRC is a potential target of Arctigenin in treating triple-negative breast cancer: based on machine learning algorithms, molecular modeling and *in Vitro* test

**DOI:** 10.3389/fmolb.2025.1644169

**Published:** 2025-09-11

**Authors:** Yuezhou Huang, Qing Luo, Linfeng Li, Tianping Li

**Affiliations:** ^1^ Department of Pharmacy, West China Hospital, Sichuan University, Chengdu, China; ^2^ Faculty of Applied Sciences, Centre for Artificial Intelligence Driven Drug Discovery, Macao Polytechnic University, Macao, China; ^3^ Sichuan Kelun-Biotech Biopharmaceutical Co., Ltd., Chengdu, China

**Keywords:** Arctigenin, triple-negative breast cancer, SRC, molecular dynamics, SPR

## Abstract

**Introduction:**

This research explores the therapeutic potential of Arctigenin (AG) against triple-negative breast cancer (TNBC) and elucidates its underlying molecular mechanisms.

**Methods:**

Potential targets of AG and TNBC-related genes were identified through public databases. By intersecting drug-specific and disease-related targets, key genes were selected for further analysis. Differential gene expression profiling and Weighted Gene Co-expression Network Analysis (WGCNA) were performed. Functional enrichment analysis was conducted using Gene Ontology (GO) and Kyoto Encyclopedia of Genes and Genomes (KEGG). Machine learning algorithms were employed to identify hub genes, followed by validation through molecular docking, molecular dynamics (MD) simulations, and surface plasmon resonance (SPR) assays. In vitro experiments including cell viability assays, cell cycle analysis, apoptosis detection, and Western blotting were performed on MDA-MB-453 and MDA-MB-231 cell lines.

**Results:**

Our study identified 183 AG-related targets, 5,193 differentially expressed genes, and 6,173 co-expression module genes associated with TNBC. Machine learning algorithms pinpointed 4 hub genes from 28 intersecting targets. Molecular docking, Molecular dynamics (MD) and surface plasmon resonance (SPR) indicated a moderately strong interaction between AG and SRC kinase, where the oxygen atom of AG forms hydrogen bonds with the oxygen atom in M341 and the nitrogen atom in G344 of SRC. In vitro experiments confirmed that AG reduced the viability of MDA-MB-453 and MDA-MB-231 cells in a concentration-and time-dependent manner, leading S phase arrest and apoptosis. Western blotting indicated that AG significantly reduced the levels of Bcl-2, caspase-3, and caspase-9, as well as decreased SRC, p-PI3K-p85, p-AKT1, p-MEK1/2, and p-ERK1/2 expression in TNBC cells in a concentration dependent manner.

**Conclusion:**

AG exerts anti-TNBC effects by directly binding to SRC kinase, concurrently inhibiting both PI3K/AKT and MEK/ERK signaling pathways, ultimately leading to cell cycle arrest and apoptosis.

## Introduction

Detection and intervention at the early stages have been identified as effective treatment options for breast cancer. However, recent reports indicate that the incidence and mortality rates of breast cancer remain high (Chlebowski et al., 2024). The International Agency for Research on Cancer reported that, in 2020, breast cancer emerged as the most prevalent type of malignant tumor, with 2.26 million new cases and an approximate mortality rate of 30% worldwide (Sung et al., 2021).

The difficulty in treating triple-negative breast cancer (TNBC) is derived from the high malignancy and recurrence rate, increasing risk of distant metastasis and mortality, as well as poor prognosis. TNBC accounts for 15%–20% of overall breast cancer incidence, commonly occurring in premenstrual women (Lin et al., 2023). Genetic profiles have demonstrated the negative expression of estrogen receptors (ERs), progesterone receptors (PRs), and human epidermal growth factor receptor 2 (HER-2) in TNBC. This molecular heterogeneity contributes to the scarcity of therapeutic targets and the insensitivity of TNBC to endocrine therapy (Karim et al., 2023). Current chemotherapies targeting TNBC are associated with a range of side effects, including cardiotoxicity, bone marrow suppression, and neurological and gastrointestinal damage, which can lead to poor patient compliance and diminished quality of life. Additionally, drug resistance often results in clinical failures, limiting the long-term use of chemotherapy (De Las Rivas et al., 2021). Hence, discovering potential inhibitors to block the proliferation and metastasis of tumor cells might shed light on TNBC treatment.

Arctigenin (AG) is one of the major bioactive ingredients extracted from the Chinese herbal medicine *Arctium lappa* L., a common spice belonging to the Asteraceae family, as identified by Japanese researchers. AG is pharmacologically functional in anti-inflammation, antiviral, anti-tumor, immunomodulation, and neuroprotection. In 1994, Hirano et al. (1994) reported the similar inhibiting bioaction of AG as classical anticancer agents on the proliferation of HL-60 cells *in vitro*, which present a relatively broad spectrum, including leukemia, prostate cancer, colon cancer, breast cancer, etc. AG exerts antitumor effects by blocking the cell cycle, inducing apoptosis and autophagy, yet low cytotoxic to normal cells, suggesting its potential as a therapeutic agent (Hyam et al., 2013; Tsai et al., 2011; Zhao et al., 2020). AG and its metabolites have garnered increasing attention in the treatment of breast cancer due to the high affinity of its phytohormone metabolites for ERs, which inhibit the bioactivity of estrogen. It could be combined with tamoxifen in the endocrine therapy of breast cancer, inducing apoptosis in tumor cells by down-regulating ERs and mTOR signaling pathways (Maxwell et al., 2018). Furthermore, studies conducted in 2020 suggested that AG induced DNA damage in HER-2 overexpressing breast cancer cells, indicating its potential efficacy against HER-2-positive breast cancer (Lee et al., 2021).

**FIGURE 1:**
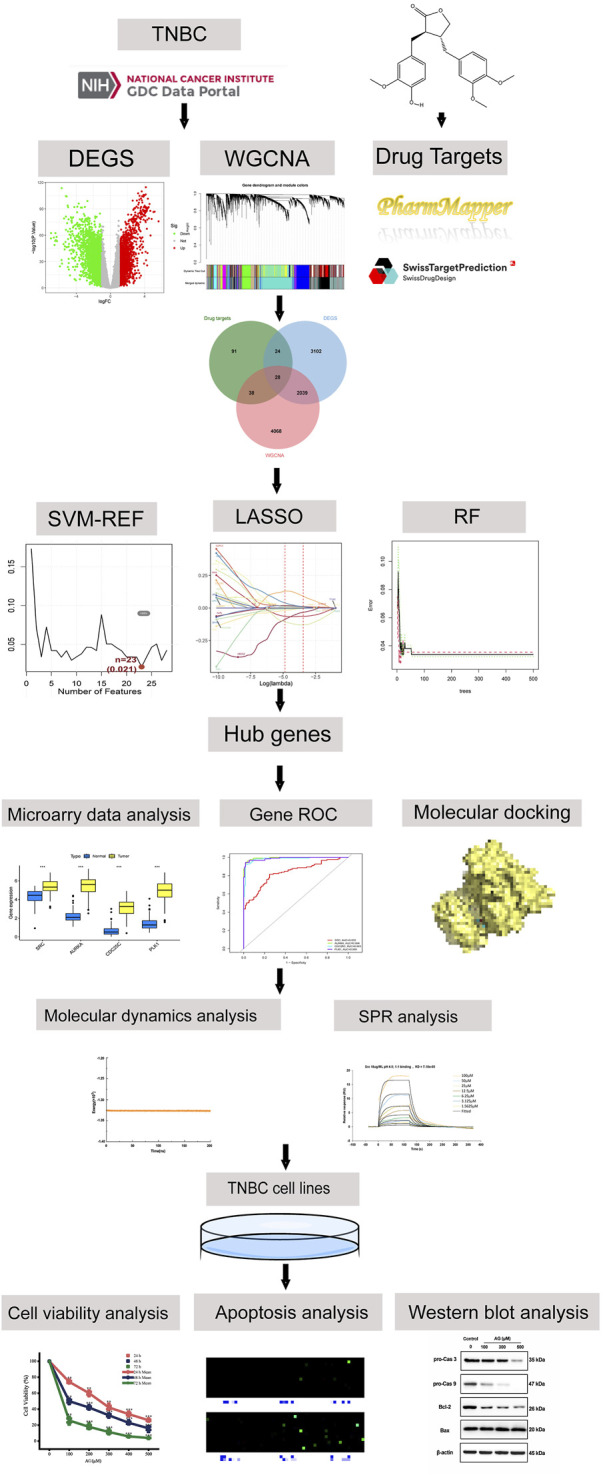
Flowchart of overall methodology used to predict the anti-cancer effect of AG for TNBC.

Network pharmacology has emerged as a robust approach to address these challenges by integrating gene expression data, molecular targets, and pathway analyses to predict compound-disease interactions across multiple biological targets (Nogales et al., 2022). This systems biology approach provides a holistic framework for identifying potential therapeutic targets and understanding the multifaceted actions of bioactive compounds. In this study, we utilized network pharmacology coupled with molecular docking to identify the key targets and mechanisms through which AG exerts its bioactivity, followed by experimental validation in human TNBC cell lines. Among the hub genes zidentified through machine learning, SRC emerged as a focal point of our investigation. SRC, plays a pivotal role in regulating key processes such as cell migration, invasion, and angiogenesis, which are critical for the aggressive nature of TNBC (Dehm and Bonham, 2004). The involvement of SRC in these processes, coupled with its established role in the progression of various malignancies, made it an ideal candidate for further exploration as a therapeutic target in the context of TNBC. The research process is shown in Figure 1.

## Materials and methods

### The databases

We have used public databases in this study and the details are listed as follows:

PubChem (https://pubchem.ncbi.nlm.nih.gov/, accessed on 10 April 2024; CID for AG: 64981).

SwissTargetPrediction (http://www.swisstargetprediction.ch/, accessed on 10 April 2024; version: 2023 release).

Pharmmapper (http://www.lilab-ecust.cn/pharmmapper/, accessed on 10 April 2024; pharmacophore model version: 2014 update).

Uniprot (https://www.uniprot.org/, accessed on 10 April 2024; database release: 2024.01).

TCGA (https://portal.gdc.cancer.gov/, accessed on 10 April 2024; project: TCGA-BRCA, data release: v36).

RCSB Protein Data Bank (PDB) (http://www.rcsb.org/, accessed on 14 April 2024; data retrieved from release archive version dated 10 April 2024).

### Acquisition of relevant targets of AG

A PubChem search was conducted using “Arctigenin” to obtain its structural formula and retrieve the SMILES. Subsequently, the MOL2 file was imported into PharmMapper and the SMILES file into the SwissTargetPrediction database to predict the corresponding target proteins of AG. After that, the acquired drug targets were annotated using the UniProt databases.

### Acquisition of AG-TNBC-related targets

Transcriptome and clinical data samples of breast cancer patients were sourced from the TCGA database, including 1,109 tumor and 113 normal breast tissue samples. After filtering samples with negative results for estrogen receptor (ER), progesterone receptor (PR), and human epidermal growth factor receptor 2 (HER2), a total of 124 TNBC samples and 113 normal samples were selected. Differentially expressed genes (DEGs) associated with TNBC were identified using the DESeq2 package in R software (version 4.1.3), applying the criteria of: |log2 fold change (FC)| ≥ 1 and adjusted p-value <0.05 (Zhu et al., 2024). The DEGs were visualized through a volcano plot and a heat map, created using the Pheatmap and ggplot2 packages, respectively.

Weighted gene co-expression network analysis (WGCNA) was performed to identify co-expression modules (Langfelder and Horvath, 2008). To enhance result robustness, the top 25% of the most significant DEGs were included in the WGCNA (Zeng et al., 2024). The expression data was first normalized through the normalizeBetweenArrays function, and genes with low variance, specifically those with a standard deviation below 0.5, were excluded (Bourgon et al., 2010). To enhance network reliability, hierarchical clustering and static tree cutting techniques were applied to remove outlier samples. The pickSoftThreshold function was then employed to assess the fit of the scale-free topology model (R^2^) and the average connectivity across various soft-thresholding powers. The ideal soft-thresholding power was determined using the criterion of R^2^ ≥ 0.9, which was applied to create a weighted adjacency matrix (Zhang and Horvath, 2005). Following this, both a weighted adjacency matrix and topological overlap matrix (TOM) were constructed, leading to hierarchical clustering and dynamic tree cutting to delineate gene modules. Modules were merged based on eigengene correlation threshold of 0.25. The correlation between module eigengenes and clinical traits was evaluated via Pearson correlation, and genes with high module membership (MM) and gene significance (GS) were retained for further analysis (Miller et al., 2011; Yip and Horvath, 2007). By using a Venn diagram to intersect DEGs, WGCNA-derived TNBC-related targets, and AG drug targets, several genes were identified as promising therapeutic candidates for TNBC treatment.

### Functional enrichment analysis

To elucidate the biological functions and key signaling pathways implicated in AG-mediated treatment of TNBC, Gene Ontology (GO) and Kyoto Encyclopedia of Genes and Genomes (KEGG) pathway enrichment analyses were performed on the overlapping target genes using the clusterProfiler package (version 4.1.3) in R software (Ashburner et al., 2000; Kanehisa and Goto, 2000). Significantly enriched terms were selected by applying a threshold of p-value <0.05 and subsequently ranked in descending order based on their enrichment scores.

### Determination of hub genes with machine learning

Three machine learning algorithms—least absolute shrinkage and selection operator (LASSO), support vector machine-recursive feature elimination (SVM-RFE), and random forest (RF)—were employed to identify hub genes among overlapping target genes (Bao et al., 2023; Sanz et al., 2018; Tang et al., 2023), aiming to selecting genes capable of distinguishing TNBC patients from healthy controls.

For SVM-RFE, a recursive feature elimination framework based on a linear kernel was executed using the e1071 package in R (Huang et al., 2018). The gene expression data underwent z-score normalization, and binary labels were assigned to the groups (Normal vs. TNBC). Feature ranking was conducted using linear SVMs, with the cost parameter set to 10 and the scaling turned off. The elimination process was directed by the squared weight coefficients. A 10-fold stratified cross-validation scheme was employed, batch elimination of 50% was applied when the number of remaining features exceeded 50. Hyperparameter optimization was performed via grid search across gamma = 2^ (−12:0) and cost = 2^ (−6:6) (Huang et al., 2017). The selection of the optimal feature subset was based on achieving the lowest classification error during cross-validation.

In our analysis using LASSO regression, we employed the glmnet package in R (v4.1.3) to construct a binomial logistic model featuring with L1 regularization (alpha = 1) (Friedman et al., 2010). Z-score normalization was applied to gene expression values, and sample groups were binarized. 10-fold cross-validation was used to identify the optimal regularization parameter (lambda.min), defined as the value yielding the lowest mean cross-validated deviance. Genes with non-zero coefficients at lambda. min were selected as potential biomarkers.

The randomForest package (Garge et al., 2013) was utilized to implement the RF model. The model was initially set up with 1,000 trees (ntree = 1,000) using the default parameters. To evaluate the model’s effectiveness, out-of-bag (OOB) error estimates were used, and the ideal number of trees was identified by finding the lowest OOB error. The significance of genes was assessed through the mean decrease in the Gini index, leading to the selection of the highest-ranked genes for further analysis.

Hub genes were identified through the intersection of results derived from three distinct machine learning algorithms. This was followed by an analysis using Receiver Operating Characteristic (ROC) curves to assess their predictive capabilities, along with correlation and differential expression studies to confirm their significance in AG interactions.

### Molecular docking

The 2D structure of AG was drawn using ChemBioDraw 2014, and its energy minimization was optimized by Chem 3D 2014. The 3D structure of the human kinase proteins were downloaded from the RCSB Protein Data Bank (PDB IDs: 4MXO, 3OP3,8JG8 and 3DB6) (Elling et al., 2008; Levinson and Boxer, 2014; Tang et al., 2024; Tsytlonok et al., 2019). Ligand preparation was performed using the Schrödinger LigPrep module (Schrödinger, LLC, New York, NY, 2018) with the OPLS_2005 force field. The docking box center was defined as the centroid of the original ligand. For the docking task, the standard precision (SP) docking mode of Glide (Friesner et al., 2004; Friesner et al., 2006; Halgren et al., 2004; Yang et al., 2021) was used with the default docking parameters.

### Molecular dynamics

Molecular dynamics (MD) was stimulated by AMBER 22 (Case et al., 2005) for the selected ligand-target complex. The AMBER14SB force field (Maier et al., 2015) was used for protein, and the force field parameters of AG were generated based on the general AMBER force field (Wang et al., 2004) by antechamber. The TIP3P water model was used in an octahedral model, with a minimum 12 Å distance between the protein surface and box boundary. Sodium were then added to neutralize the system. Each simulation system was initially energy-minimized to optimize unreasonable atomic contacts and stereochemical conflicts by applying position restraints (force constant of 5.0, 1.0, and 0 kcal·mol^−1^·Å^−2^, respectively) on the backbone atoms and AG. Subsequently, the solvent was heated to 300 K in 50 ps with all solute atoms restrained with a force constant of 10.0 kcal·mol^−1^·Å^−2^. Next, two 50 ps equilibration steps were done in the NPT ensemble with temperature and pressure (1 bar) control by the Langevin thermostat and Berendsen barostat method. Periodic boundary conditions were applied to eliminate boundary effects. All solute atoms were restrained with force constants of 1.0 and 0.5 kcal·mol^−1^·Å^−2^. The production run, with no restraints, was performed for 200 ns. Electrostatic interactions were calculated by the particle mesh Ewald (PME) (Poier et al., 2023). The cutoff distance for nonbonded interactions was 8 Å. The SHAKE algorithm was used to constrain bonds involving hydrogens. The integration time step was set to 2 fs, and conformations were sampled every 10 ps for subsequent analysis.

### MM/GBSA calculations

To acquire more statistically significant results, the binding free energy between SRC and AG was calculated for the 50 ns of MD trajectories every 0.5 ns via the conventional MM/GBSA approach in AMBER tools according to procedures in our previous work (Guo et al., 2012). For each frame, the free energy was calculated for each molecular species (SRC-AG complex, SRC, and AG), and the binding free energy was computed as below (Chong et al., 2009; Kollman et al., 2000):

$$\Delta G_{bind}=G_{complex}-\left(G_{receptor}+G_{ligand}\right)$$ (1)

$$\Delta G_{bind}=\Delta H-T\Delta S\approx \Delta G_{gas}+\Delta G_{sol}$$ (2)

$$\Delta G_{gas}=\Delta E_{MM}-T\Delta S$$ (3)

$$\Delta E_{MM}=\Delta E_{\mathrm{int}}+\Delta E_{ele}+\Delta E_{vdw}$$ (4)

$$\Delta G_{sol}=\Delta G_{pol,sol}+\Delta G_{npol,sol}$$ (5)

where $\Delta G_{bind}$ (Equations 1, 2) denotes the binding free energy, and it can be decomposed into two terms: (1) The free energy in a vacuum, $\Delta G_{gas}$ is decomposed into the molecular mechanical energy ( $\Delta E_{MM}$ ) and the configurational entropy ( $\left.-T\Delta S\right)$ (Equation 3). $\Delta E_{MM}$ is the summation of the intramolecular energy ( $\Delta E_{\mathrm{int}}$ , including bond, angle, and dihedral energies, which is 0 in this study), electrostatic energy ( $\Delta E_{ele}$ ), and van der Waals energy ( $\Delta E_{vdw}$ ) (Equation 4). The entropic contribution ( −TΔS ), which is associated with the conformational entropy loss when a free-state ligand binds to the corresponding unbound-state receptor; (2) the solvation energy ( $\Delta G_{sol}$ ), which is composed of the polar ( $\Delta G_{pol,sol}$ ) and non-polar contributions ( $\Delta G_{npol,sol}$ ) (Equation 5).

### Surface plasmon resonance (SPR)

SRC protein (MedChemExpress, New Jersey, United States) was diluted to 2 μg/mL with acetate solutions at pH4.5, pH5.0, and pH5.5, respectively. The protein was immobilized on the chip with acetate solutions at pH4.5 and a concentration of 18 μg/mL. One channel of SRC was immobilized before coupling the ligand, the chip was activated for 420 s. The coupling was stopped when the amount reached 13800 RU. Ligand coupling was completed after blocking the chip for 420 s. The actual immobilized amount was 11700 RU. Analyte AG was prepared as the solution with concentrations of 800, 400, 200, 100, 50, 25, 12.5, 6.25, 3.125, and 1.5625 μM by gradient dilution. Multi-cycle kinetics runs were performed with a period of association of 120 s and dissociation of 240 s. The buffer contained 1xPBS (pH 7.4), 0.05% Tween-20, and 5% DMSO. The raw data was imported into BiacoreTM Insight Evaluation Software 4.0, and the multi-cycle kinetics evaluation method was selected for calculating kinetic rate constants. The curve was fitted by the 1:1 binding model with data from 5 chosen concentrations. The association rate constant (K_a_), dissociation constant (K_d_), and affinity constant (KD) were acquired.

### Cell culture and treatment

MDA-MB-453 cell line and MDA-MB-231 cell line purchased from the National Infrastructure of Cell Line Resource (NICR, Beijing, P.R. China). The cells lines were cultured in RPMI-1640 medium, supplemented with 10% (v:v) of fetal bovine serum (FBS; Biological Industries, Kinneret, Israel) and 1% penicillin-streptomycin solution (Hyclone|Cytiva, Marlborough, United States) at 37 °C in a 5% CO_2_ incubator.

### Cell viability assay

Cell viability was evaluated by Cell Counting Kit-8 (CCK8 assay; MedChemExpress, New Jersey, United States). MDA-MB-453 and MDA-MB-231 cells were seeded into 96-well plates (3 × 10^4^ per well) and pre-cultured for 24 h, then incubated in 200 μL complete medium containing AG (0, 100, 200, 300, 400, and 500 μM) for 24 h, 48 h or 72 h. Subsequently, CCK8 solution was added to each well and incubated in the dark at 37 °C for 1 h. The absorbance was measured at 450 nm on a microplate reader (Eon Microplate Spectrophotometer, BioTek, United States). All experiments were repeated thrice independently. Cell viability (%) = $\left(A_{450}\text{ of drug}-A_{450}\text{ of blank}\right)/\left(A_{450}\text{ of control }–\;A_{450}\text{ of blank}\right)\times 100\;\%$ .

### Cell cycle analysis

The cell cycle was assessed using a PI staining kit (KeyGEN BioTECH, Nanjing, P.R. China) following the manufacturer’s instructions via flow cytometry. After incubating with PI/RNase A for 45 min in the dark, the cells were conducted using a flow cytometer (Cytoflex, Beckman, United States). Software Modfit LT was employed for cell cycle distribution analysis. A total of 20,000 events from each cell sample were obtained. All experiments were repeated thrice independently.

### Caspase-3 activity analysis

GreenNuc™ caspase-3 Assay Kit for Live Cells (Beyotime, Shanghai, P.R. China) was subjected to caspase-3 activity analysis following the manufacturer’s instructions. After incubation at room temperature for 30 min, a fluorescence microscope (OBSERVER D1/AX10 cam HRC, Zeiss, Germany) was used to observe green fluorescence.

### Mitochondrial membrane potential (MMP) analysis

MMP assay kits with JC-1 (Beyotime, Shanghai, P.R. China) were purchased to detect MMP of MDA-MB-453. The fluorescence signals of AG-treated or untreated cells were detected via flow cytometry. All experiments were repeated thrice independently.

### Apoptosis analysis

Apoptosis was assessed by an annexin V-FITC/PI staining kit (KeyGEN BioTECH, Nanjing, P.R. China) following the manufacturer’s instructions. After incubating with 5 μL Annexin V-FITC and 5 μL PI for 15 min in the dark, the cells were conducted using a flow cytometer (Cytoflex, Beckman, United States) and analyzed by software FlowJo V10. A total of 20,000 events from each cell sample were obtained. All experiments were repeated thrice independently.

### Western blot analysis

Total proteins were lysed from AG-treated cells (100, 300, and 500 μM) or untreated cells in RIPA buffer with the protease inhibitor cocktail and PMSF (Beyotime, Shanghai, P.R. China). The concentration of proteins in each group was tested by the BCA Protein Assay Kit (Beyotime, Shanghai, P.R. China). Protein extracts were quantitated and loaded on 8%–12% sodium dodecyl sulfate-polyacrylamide gel, then electrophoresed and transferred onto a PVDF membrane (Beyotime, Shanghai, P.R. China), which was blocked in 5% skimmed milk for an hour. The membranes were incubated with primary antibody overnight at 4 °C. The primary antibodies used were anti-caspase-3, anti-caspase-9, anti-Bax, anti-Bcl-2, anti-CDK2, anti-cyclin A2, Anti-P27, anti-SRC, anti-AKT1, anti-pAKT1, anti-ERK1/2, anti-pERK1/2, anti-PI3K(p85), anti-p-PI3K(p85), anti-MEK1/2, anti-p-MEK1/2and anti-β-actin antibodies (human anti-rabbit, 1:1,000; CST, Massachusetts, United States). Then, the membranes were washed and incubated with horseradish peroxidase (HRP)-conjugated secondary antibody (Goat anti-rabbit, 1:100; ZSGB-BIO, Beijing, P.R.China) for 1 h. The positive signal on the membranes was detected by an enhanced chemiluminescence detection kit (Beyotime, Shanghai, P.R. China). Band intensities were scanned and quantified by NIH ImageJ software.

### Statistical analysis

Data were presented as the mean ± SD. Differences between data groups were evaluated using the one-way analysis of variance followed by the Dunnett test, using GraphPad Prism 8 software. P < 0.05 was considered as a statistically significant result.

## Result

### Screening of target predictions

**FIGURE 2:**
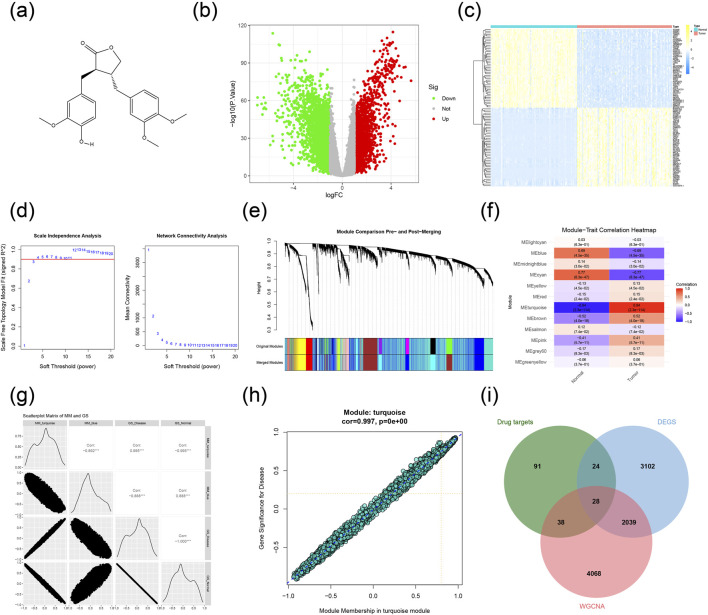
Construction of target network and acquisition of key genes. **(a)** Structural formula of AG. **(b)** Differential expression analysis of the TCGA dataset. **(c)** Heatmap of DEGs showing the top 50 genes. **(d)** Scale independence and mean connectivity of WGCNA. **(e)** Cluster dendrogram and separation of gene modules of WGCNA. **(f)** Diagram of module-trait relationship for the 12 modules. **(g)** Scatterplot matrix of MM and GS. **(h)** catterplot of GS for TNBC vs. MM of the turquoise module. **(i)** Key genes for the action of AG.

The molecular structure is depicted in Figure 2a, and a total of 183 potential targets for AG were identified on the PharmMapper and Swiss Target Prediction database (Supplementary Table S1). Differential expression analysis of the TCGA dataset revealed 5193 DEGs, which were visualized using a heatmap and a volcano plot (Figures 2b,c; Supplementary Table S1). To explore the molecular mechanisms underlying TNBC, we constructed a gene co-expression network via WGCNA. The scale-free topology fit index (R^2^) and mean connectivity were assessed with the pickSoftThreshold function across a range of powers (β = 1–20). As shown in Figure 2d, the R^2^ value surpassed the recommended threshold of 0.90 at a power of 3, while maintaining acceptable mean connectivity, justifying its selection as the ideal soft-threshold for network construction. Genes were clustered and partitioned into modules using the dynamic tree cut method (Figure 2e), resulting in 12 distinct gene modules, with the cluster dendrogram shown in Figure 2f. Additionally, a network heatmap was created to visualize the correlations among genes within each module (Figure 2g). Subsequently, the analysis of module-trait relationships indicated that the turquoise module had the strongest association with tumor/normal control phenotypes (Figure 2h), showing a significant positive correlation (cor = 0.997) between gene significance for TNBC and module membership (Figure 2i). These results suggest that the 6173 genes in the turquoise module are associated with TNBC and may serve as a reservoir of candidate genes for further prioritization (Supplementary Table S1). By intersecting the DEGs, WGCNA key module genes, and the predicted targets of AG, we identified 28 overlapping genes (Figure 2j; Supplementary Table S1), proposed as potential therapeutic targets for AG in TNBC treatment.

### GO enrichment and KEGG pathway analysis

**FIGURE 3:**
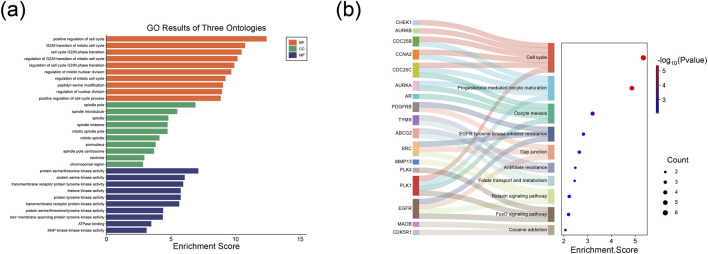
Functional enrichment analysis of key genes in TNBC. **(a)** Bar plot from the GO analysis. **(b)** Sankey-bubble plot from the KEGG analysis.

We performed GO and KEGG pathway enrichment analyses on these 28 intersecting target genes. The GO analysis results are presented in Figure 3a. The potential target genes were predominantly enriched in the following biological process (BP) terms: positive regulation of the cell cycle, G2/M transition of mitotic cell cycle, cell cycle G2/M phase transition, regulation of G2/M transition of mitotic cell cycle, regulation of nuclear division, and regulation of the cell cycle G2/M phase transition. Regarding cellular components (CC), the enriched entries included the spindle pole, spindle microtubule, spindle, spindle midzone, and mitotic spindle pole. For molecular functions (MF), the enriched terms included protein serine/threonine kinase activity, protein serine kinase activity, transmembrane receptor protein tyrosine kinase activity, histone kinase activity, and protein tyrosine kinase activity. In addition, based on the KEGG pathway enrichment analysis, these intersecting target genes were mainly enriched in signaling pathways such as the Cell cycle, Progesterone-mediated oocyte maturation, Oocyte meiosis, EGFR tyrosine kinase inhibitor resistance, and Gap junction (Figure 3b).

### Determination of target hub genes with machine learning

**FIGURE 4:**
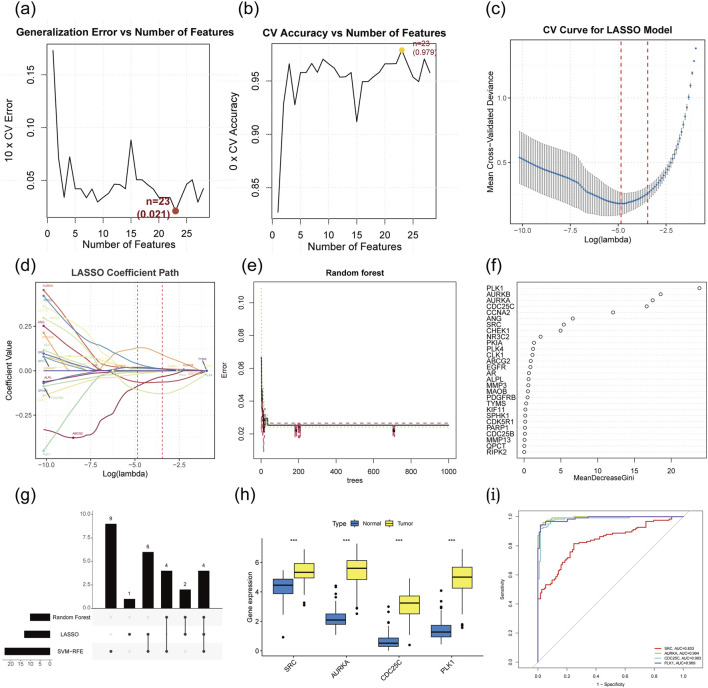
Determining target hub genes through machine learning algorithms. **(a)** Error rate curves of 5-fold cross-validation of SVM-RFE algorithm. **(b)** Accuracy rate curves of 5-fold cross-validation of SVM-RFE algorithm. **(c)** Coefficients diagrams of Lasso analysis. **(d)** Cross validation curve for Lasso. **(e)** Error rate curve of RF method. **(f)** Variable importance value of RF method. **(g)** Hub genes identification from three machine learning algorithms. **(h)** Expression analysis of the hub genes based on the TCGA dataset. **(i)** ROC curve of hub genes.

To further determine the critical hub genes in TNBC treatment using AG, we set the capability to discriminate between TNBC samples and non-TNBC samples in the TCGA dataset as the evaluation criterion and filtered the 28 intersecting target genes using three machine learning algorithms. We narrowed down 28 overlapping target genes through the application of three machine learning techniques (Supplementary Table S2). The SVM-RFE method revealed 23 core target genes (Figures 4a,b). Meanwhile, LASSO analysis highlighted 13 of the 28 genes as significant core targets (Figures 4c,d). The RF algorithm provided variable importance scores for all potential target genes, leading to the identification of 10 core genes based on these scores (Figures 4e,f). By computing the intersection of these machine-learning-predicted core target genes, 4 genes (AURKA, SRC, PLK1, and CDC25C) were identified as the target hub genes for TNBC treatment with AG (Figure 4g). Subsequently, we conducted gene expression analyses on different samples for these five target hub genes (Figure 4h). The results showed that these genes are closely interrelated, with their expression levels significantly higher in TNBC tissues compared to non-TNBC tissues. To explore the diagnostic efficacy of the 4 hub genes, ROC curve analysis was performed, with hub genes exhibiting an AUC value >0.7 considered diagnostic markers. In the TCGA dataset, the AUC values were 0.833 for SRC, 0.994 for AURKA, 0.983 for CDC25C, and 0.989 for PLK1 (Figure 4i).

### The keys targets of the AG-mediated

**FIGURE 5:**
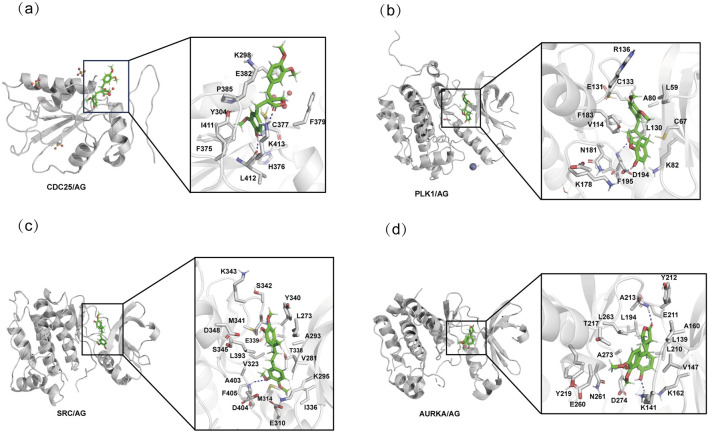
Molecular docking interactions of AG with hub genes. **(a)** Molecular docking interaction between AG and CDC25. **(b)** Molecular docking interaction between AG and PLK1. **(c)** Molecular docking interaction between AG and SRC. **(d)** Molecular docking interaction between AG and AURKA.

Molecular docking analysis was performed to validate the interaction between AG and the identified hub genes, with the most prominent binding interactions visualized in Figures 5a-d. According to established criteria, ligand-receptor binding affinities are considered biologically significant when the binding energy falls below −5 kcal/mol. Remarkably, AG demonstrated strong binding affinity with three critical targets: SRC (−7.777 kcal/mol), PLK1 (−7.690 kcal/mol), and AURKA (−7.685 kcal/mol), as detailed in Table 1. By integrating both docking scores and Glide emodel scores, SRC emerged as the most promising therapeutic target and was therefore prioritized for experimental validation. To validate the docking protocol, known SRC inhibitors were redocked, and the root mean square deviation (RMSD) values of the redocked structures compared to their original conformations in the protein data bank (PDB) were calculated. The RMSD values for PDB IDs: 1Y57, 2H8H, 3EL8, and 4MXO were found to be 1.94 Å, 1.08 Å, 0.80 Å, and 2.50 Å, respectively (Supplementary Table S3). These RMSD values can be used to assess the reliability of the docking method; generally, lower RMSD values (considered reliable when less than 2 Å, though it depends on the specific research system) indicate that the docking results are relatively credible, providing a methodological validation basis for subsequent molecular docking.

**TABLE 1:** Molecular docking results of AG with hub genes (kcal/mol).

Proteins	Docking score	XP Gscore	Glide gscore	Glide emodel
CDC25	−3.485	−3.487	−3.487	−40.785
SRC	−7.777	−7.779	−7.779	−59.389
PLK1	−7.690	−7.692	−7.692	−59.150
AURKA	−7.685	−7.686	−7.686	−57.497

**FIGURE 6:**
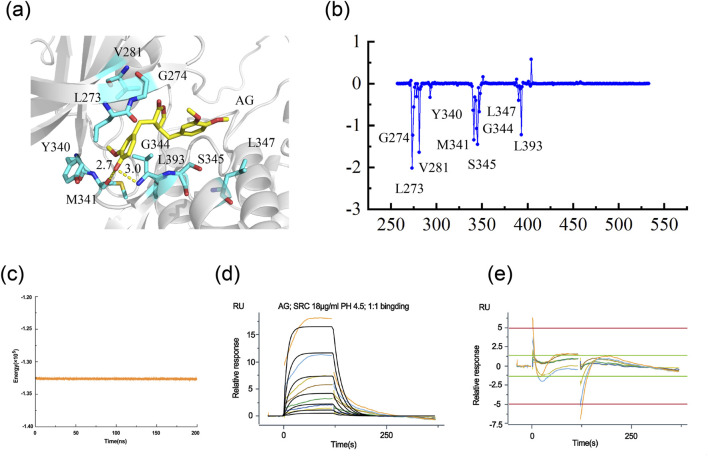
The binding mode of AG and SRC. **(a)** 3D binding mode of Arctigenin and 4MXO. **(b)** Analysis revealed specific key residues, per-residue contribution less than −1 kcal/mol. **(c)** RMSF analysis of AG with SRC. **(d)** Full SPR Sensorgrams of Src Protein Binding to AG. **(e)** Fitting Residuals of Src Protein Binding to AG.

To investigate the binding pattern of SRC and AG, MD simulations and SPR were conducted. The overall binding mode is shown in Figure 6a. The oxygen atom of AG forms hydrogen bonds with the oxygen atom in the M341 and the nitrogen atom in the G344 of SRC. The formation of these hydrogen bonds may influence the affinity of AG to SRC, thereby affecting its biological activity. The binding free energy provided showed the relative binding strengths of SRC-AG complex (Table 2). Using the MM/GBSA methods, the ΔEvdw was calculated to be −34.85 ± 5.19 kcal/mol for the SRC-AG complex, which contributed to the main part. On the other hand, the electrostatic energy was calculated to be −17.97 ± 5.35 kcal/mol. The total free binding energy was calculated to be −26.66 ± 3.75 kcal/mol for the complex. The binding free energy decomposition analysis identified key residues, as shown in Figures 6b,c. The analysis revealed specific key residues (per-residue contribution less than −1 kcal·mol^−1^) (7 residues), highlighting the role of amino acids such as G274, V281, M314, S345, G344, and L393 in recognizing AG.

**TABLE 2:** The contributions of each energy term to the binding energy of AG with SRC (kcal/mol).

Energy Component	ΔE_vdw_	Δ_Eele_	ΔG_pol,sol_	ΔG_npol,sol_	ΔG_gas_	ΔG_sol_	Δ*G*_MM/GBSA_
Contributions	−34.85 ± 5.19	−17.97 ± 5.35	30.74 ± 2.90	−4.58 ± 0.75	−52.82 ± 0.75	26.16 ± 3.64	−26.66 ± 3.75

For a deeper investigation of the interaction between AG and SRC, SPR-based binding analysis was performed. The full SPR sensorgrams and corresponding fitting residuals are shown in Figures 6d,e, respectively. A 1:1 Langmuir binding model was employed for kinetic fitting, with a Chi-squared (χ^2^) value of 0.604 RU^2^ indicating a good fit. The kinetic parameters, detailed in Table 3, revealed that AG binds to SRC with an equilibrium dissociation constant (KD) of 71.8 μmol/L, an association rate constant (ka) of 6.95 × 10^2^ M^−1^·s^−1^, and a dissociation rate constant (kd) of 4.99 × 10^−2^ s^−1^. These values suggest a moderately strong binding affinity and validate the specific interaction between AG and SRC.

**TABLE 3:** SPR kinetic parameters for the interaction between immobilized SRC and AG.

Immobilized ligand	Injection variables Analyte 1 solution	Quality kinetics Chi^2^ (RU^2^)	1:1 binding ka (1/Ms)	kd (1/s)	KD (M)
SRC 18 μg/mL	AG	6.04e−01	6.95e + 02	4.99e−02	7.18e−05

### *In vitro* validation of AG treatment for TNBC

**FIGURE 7:**
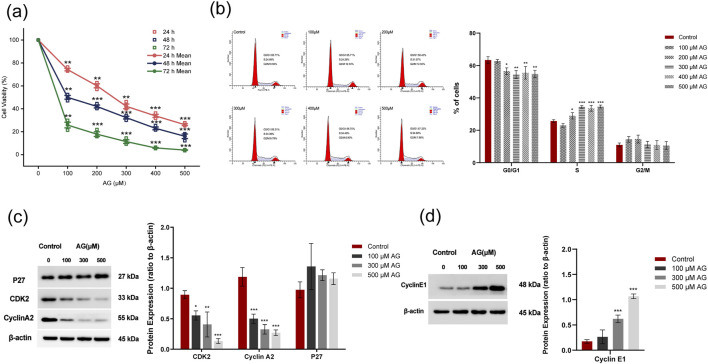
Effects of AG on the proliferation of MDA-MB-453 cells. **(a)** Cell viability was determined by CCK8 assay. **(b)** Cell cycle changes were analyzed by FACS based on PI staining. **(c)** Cells were incubated with various concentrations of Arctigenin for 48 h and tested the expression of CDK2, Cyclin A2, and P27 by Western blot. **(d)** Expression of Cyclin E1 examined by Western blot. $\bar{x}$ ± *s*, *n* = 3, *, ** and *** indicate 0.01 < *P* < 0.05, *P* < 0.01 and P < 0.001 vs. untreated control.

The cell viability of MDA-MB-453 cells incubated with a series of concentrations of AG for 12 h, 24 h, and 48 h was examined (Figure 7a). Compared with the control, the viability of AG-treated cells was negatively correlated with the concentrations of AG, as well as the duration of incubation. The AG-treated group showed a low G0/G1 ratio and an increased ratio of S-phase cells. No significant change was observed in the ratio of G2/M phase cells (Figure 7b), suggesting AG might induce cell cycle arrest to inhibit the proliferation of tumor cells by blocking the process of DNA replication. Similar inhibitory effects were noted in MDA-MB-231 cells as seen in MDA-MB-453. A marked decline in cell viability was observed with higher AG concentrations and extended treatment durations (Supplementary Figure S1a). Cell cycle analysis revealed that AG treatment led to a decrease in the G0/G1 phase cell population while increasing the S phase, with no significant changes in the G2/M phase (Supplementary Figure S1b). Expression of cyclins, CDK2, and Cyclin A2 decreased, while the level of P27 showed no significant change, suggesting AG-induced DNA replication arrest was CDK2/Cyclin A2-targeted (Figure 7c). Interestingly, we also found increased Cyclin E1 expression after AG treatment, which might have accounted for apoptosis (Figure 7d).

**FIGURE 8:**
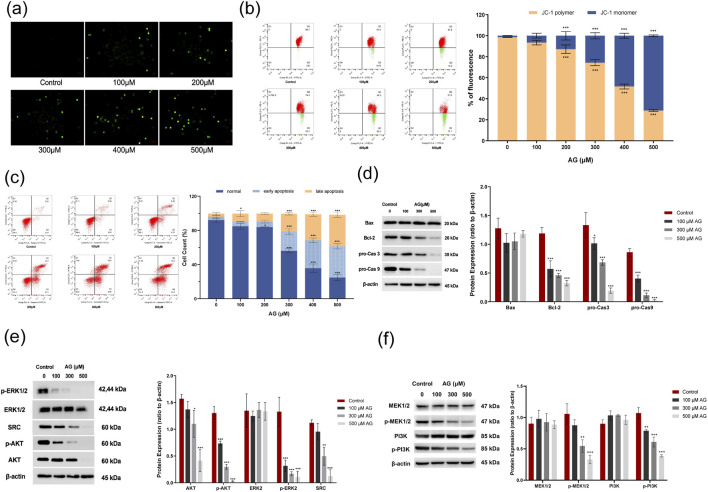
Effects of AG on the apoptosis of MDA-MB-453 cells. **(a)** Cells were incubated with various concentrations of AG for 48 h and stained with fluorescent dye. **(b)** MMP changes were analyzed by FACS based on JC-1 staining. **(c)** Apoptosis rate was analyzed by FACS based on Annexin-FITC/PI staining. **(d)** Expression of Bax, Bcl-2, caspase-3, and caspase-9 using western blot. **(e)** Impact of AG on the expression of ERK1/2, p-ERK1/2, AKT, p-AKT and SRC. **(f)** Impact of AG on the expression of PI3K, p-PI3K, MEK1/2 and p-MEK1/2. $\bar{x}$ ± *s*, *n* = 3, *, ** and *** indicate 0.01 < *P* < 0.05, *P* < 0.01 and P < 0.001 vs. untreated control.

To verify the AG-induced cytotoxicity, MDA-MB-453 cells were employed to apoptosis indications after AG incubation for 48 h. Detected MMP showed depolarization, andcaspase-3 activity was promoted, which both were AG concentration-dependent, suggesting the apoptosis event was AG-relevant (Figures 8a,b). The Annexin-FITC/PI-staining flow cytometry results showed that AG treatment increased death rate in a concentration-dependent manner (Figure 8c). It is worth noting that the number of cells treated with low concentrations of AG showed no significant differences in early apoptosis (100 and 200 μM) but mostly varied in late apoptosis or necrosis. Apoptosis-related protein indicators showed that Bcl-2, pro-caspase-9, and pro-caspase-3 were downregulated, yet cleaved caspase-9 and cleaved caspase-3 increased in a concentration-dependent manner (Figure 8d). In MDA-MB-231 cells, the evaluation of caspase-3 activity showed a positive correlation between the number of apoptotic cells and AG concentration (Supplementary Figure S1c). Further confirmation from Annexin V-FITC/PI staining flow cytometry indicated that AG treatment also increased apoptosis in a concentration-dependent manner (Supplementary Figure S1d). Apoptosis-related protein analysis showed significant downregulation of Bcl-2, pro-caspase-9, and pro-caspase-3 with increasing AG concentrations, while Bax expression remained unchanged between treatment and control groups (Supplementary Figure S1e). The results suggested AG might trigger intracellular caspase cascades. Combined with depolarized MMP, AG-induced apoptosis in TNBC cell lines might be achieved via a caspase-dependent mitochondrial apoptosis pathway.

To investigate the AG-mediated SRC-related protein expression, MDA-MB-453 cells were incubated with AG for 48 h. The results showed that the levels of t-AKT and p-AKT decreased. The level of p-ERK1/2 attenuated, hence indicating an entirely decreased ERK1/2 phosphorylation in the cytosol and effective AG-evoked blockage on ERK1/2 activation (Figure 8e). Meanwhile, as shown in Figure 8f, there were no notable changes in the levels of PI3K and MEK1/2 following AG treatment compared to the control group, while p-PI3K and p-MEK1/2 levels were markedly lower. A decrease in SRC levels was also noted, suggesting that AG modulates the PI3K-AKT and MEK/ERK pathways by downregulating SRC expression. Furthermore, we assessed the expression of SRC, AKT, p-AKT, ERK1/2, p-ERK1/2, MEK1/2, p-MEK1/2, PI3K, and p-PI3K in MDA-MB-231 cells. The results were largely consistent with those observed in MDA-MB-453 cells, showing significant reductions in SRC, p-PI3K, p-AKT, p-MEK1/2, and p-ERK1/2 in the AG-treated groups (Supplementary Figures S1f,g).

## Discussion

AG has garnered significant attention for its broad therapeutic potential in treating various human diseases, owing to its diverse pharmacological properties, including anti-inflammatory, antiviral, anti-tumor, neuroprotective, and immunomodulatory effects (Wu et al., 2022). Notably, AG exhibits remarkable inhibitory effects on breast cancer cells, inducing cell cycle arrest, apoptosis, or autophagy, and suppressing cancer cell metastasis (Feng et al., 2017; Shabgah et al., 2021; Zhu et al., 2020). Importantly, ER-negative breast cancer cells demonstrate greater sensitivity to AG compared to ER-positive cells (Hsieh et al., 2014), suggesting its potential utility in TNBC treatment. However, the precise molecular targets and binding mechanisms of AG in TNBC remain poorly understood. In this study, we utilized an integrative approach combining network pharmacology, molecular docking, molecular dynamics (MD) and surface plasmon resonance (SPR) to elucidate the effects of AG on TNBC, with experimental validation in MDA-MB-453 and MDA-MB-231 cells.

Through analysis of public databases, we identified 183 potential drug targets of AG. Further examination of tumor and adjacent non-tumor tissues from TNBC patients revealed 5193 DEGs and 6137 co-expressed module genes. The integration of these datasets led us to identify 28 significant genes, which were then analyzed through GO and KEGG enrichment assessments. These analyses highlighted the enrichment of AG’s mechanism of action in cell cycle regulation, underscoring its potential to disrupt TNBC progression. Using three machine-learning algorithms, we identified four hub genes-SRC, AURKA, PLK1, and CDC25-which exhibited high expression in tumor tissues and area under the receiver operating characteristic curve (AUC) values exceeding 0.8, suggesting their potential as therapeutic targets and prognostic markers for TNBC. AURKA and PLK1 are essential mitotic regulators that promote the G2/M transition and are often overexpressed in aggressive breast cancers (D'Assoro et al., 2015; Wang D. et al., 2017), while CDC25C facilitates CDK1 activation and has been associated with unchecked cell cycle progression in high-grade tumors (Topno et al., 2021). Additionally, SRC as a non-receptor tyrosine kinase involved in migration, proliferation, and chemoresistance, and its hyperactivation is characteristic of metastatic and drug-resistant TNBC phenotypes (Finn et al., 2011; Kohale et al., 2022). Collectively, these genes are involved in cell cycle and survival pathways, suggesting that this gene module forms a biologically relevant and therapeutically viable network, potentially mediating the effects of AG in TNBC.

Additionally, we investigated a group of genes identified by at least two of the three analytical models, which we will refer to as near miss candidates. These genes warrant particular focus due to their developing functional importance in triple-negative breast cancer (TNBC) and their possible connection to AG’s mechanism. Both SVM RFE and LASSO consistently highlighted CLK1 and PDGFRB. CLK1 is known to influence the alternative splicing of genes involved in the cell cycle and is often overexpressed in breast cancer; its pharmacological inhibition can disrupt splicing and hinder tumor growth (Liu et al., 2025; Zhu et al., 2018). PDGFRB signaling in the tumor microenvironment promotes epithelial-mesenchymal transition (EMT) and is inhibited by BRCA1, making it a significant therapeutic target, particularly in BRCA1-deficient TNBC (Bai et al., 2021). MMP13 and RIPK2 also appeared in the SVM RFE and LASSO intersection. MMP13 and RIPK2 were also found in the overlap of SVM RFE and LASSO. MMP13 is associated with bone metastasis and osteolytic processes in breast cancer, indicating its potential as a therapeutic target (Zhu et al., 2023). Increased levels of RIPK2 are linked to unfavorable outcomes in TNBC and facilitate tumor advancement through the activation of the NF-κB and JNK pathways (Jaafar et al., 2018; Singel et al., 2014). CHEK1, PKIA, and NR3C2 were identified by both SVM-RFE and Random Forest or by Random Forest and LASSO. Importantly, CHEK1 is involved in the DNA damage response and has been considered a target for TNBC treatment (Gatti-Mays et al., 2020). Although PKIA and NR3C2 are not well understood in the context of TNBC, emerging data from other cancer types indicate they may have important roles in kinase signaling and tumor biology, necessitating further functional studies (Liu et al., 2023; Steegmaier et al., 2007; Xu B. et al., 2023). ANG was identified by both LASSO and Random Forest; its expression increases under low oxygen conditions, aiding in angiogenesis and tumor survival, with studies showing that its inhibition can reduce breast cancer growth *in vivo* (Chintalapati et al., 2009). Collectively, these near-miss genes form a biologically significant group with potential functional implications for TNBC development and AG’s mechanism of action. Although they were not part of the final hub gene intersection, their repeated identification across various feature selection methods indicates their reliability, suggesting they could be important targets for subsequent functional validation.

To further investigate the drug targets of AG in the treatment of TNBC, molecular docking revealed strong binding affinities between AG and SRC, AURKA, and PLK1 (Glide scores < −7), while its interaction with CDC25 was weaker (Glide score > −5). Based on the Glide emodel score, we hypothesized that the AG-SRC interaction plays a pivotal role in TNBC regulation. MD simulations further elucidated the binding mode of AG to SRC, revealing a van der Waals energy (ΔEvdw) of −34.85 ± 5.19 kcal/mol for the SRC-AG complex. Critical amino acid residues, including G274, V281, M314, S345, and G344, were identified as key interaction sites.

The selection of a 200 ns simulation timescale in this study was primarily guided by our goal of elucidating ligand binding modes and characterizing key protein-ligand interactions, rather than determining precise kinetic parameters. Although enhanced sampling techniques, such as WESTPA or metadynamics, may offer advantages for sampling rare events, our approach, which uses multiple independent conventional MD trajectories, has proven effective for mapping binding sites and identifying interaction patterns in similar kinase systems. Previous studies have shown that simulation timescales ranging from 200 to 500 ns are effective for identifying ligand binding sites and characterizing interaction patterns (Alanzi et al., 2024; Sulaimani et al., 2025). Our approach compensates for the shorter timescale by employing multiple independent trajectories and ensuring comprehensive conformational sampling. The results exhibit good convergence of structural metrics and show excellent agreement with experimental binding modes. In our MM/GBSA calculations, we elected to exclude the entropy term (–TΔS) based on established limitations in obtaining accurate entropy estimates for complex biomolecular systems (Ruvinsky, 2007). While this results in reported ΔG values (−26.66 ± 3.75 kcal/mol) that strictly represent enthalpic contributions (ΔH), this methodological choice does not compromise our principal conclusions for several compelling reasons: (1) Entropic effects typically exhibit systematic behavior across structurally similar ligand, preserving the validity of relative binding affinity comparisons; (2) Our analytical focus centers on structural interaction patterns rather than absolute free energy quantification; and (3) Available literature provides well-characterized benchmarks for entropy contributions in small molecule-protein binding events (6–15 kcal/mol), enabling appropriate interpretation when required (Chang et al., 2007).

SRC, a non-receptor tyrosine kinase, is a critical regulator of cell proliferation, migration, and apoptosis (Chen et al., 2019; Patel et al., 2016; Ramadan et al., 2021). Under normal physiological conditions, SRC activity is tightly controlled to maintain cellular functions such as adhesion, survival, and angiogenesis (Le and Bast, 2011). However, SRC is overexpressed in various solid tumors, including breast, pancreatic, gastric, and bladder cancers, where it accelerates tumor cell growth and survival (Luo et al., 2022; Su et al., 2023; Wang et al., 2022; Xu et al., 2021). In this study, AG exhibited moderate binding affinity to SRC (KD = 71.8 μmol/L), a notable value for an unmodified natural compound (Wang X. et al., 2017), indicating a moderate binding affinity between AG and SRC. The relatively low association rate constant and reversible dissociation rate suggested that AG interacts with SRC in a specific yet dynamically regulated manner. This level of affinity allows AG to regulate SRC activity without causing permanent inhibition, a characteristic that is often beneficial in the modulation of signaling pathways (Csermely et al., 2005; Lu and Tonge, 2010).

Mechanistically, SRC influences cell cycle progression by phosphorylating cyclins and cyclin-dependent kinases (CDKs) (Zhang et al., 2006). CDK2, in complex with Cyclin A or Cyclin E, regulates distinct phases of the cell cycle. The CDK2/Cyclin A complex facilitates DNA synthesis during the S phase and prepares chromosomes for division in the G2 phase (Tsytlonok et al., 2019), while the CDK2/Cyclin E complex promotes the G1-to-S transition by initiating DNA synthesis-related gene expression and inhibiting the cell cycle inhibitor p27 (Lai et al., 2016). In our study, AG treatment downregulated CDK2 and Cyclin A2, leading to S-phase arrest in MDA-MB-453 cells, suggesting impaired DNA synthesis.

It has been reported that persistently phosphorylated or overexpressed SRC kinase leads to pathological modulation of several tumor cells proliferation-relevant signaling, namely PI3K/AKT pathway, MEK/ERK pathway, and JAK/STAT3 pathway (Ferguson et al., 2013; Rodriguez Torres et al., 2023; Xu R. et al., 2023). By enhancing PI3K activity and AKT phosphorylation, SRC promotes cell proliferation and survival while influencing the tumor microenvironment to support tumor growth and metastasis (Ye et al., 2025). In breast cancer cells, SRC-driven PI3K/AKT activation is a critical driver of cell apoptosis (Luo et al., 2020). Maxwell et al. identified that AKT, NF-κb, and MAPK pathways were involved in AG-relevant anti-cancer effects in (either ER-positive or ER-negative) breast cancer (Maxwell et al., 2017). Consistent with this, AG treatment reduced the phosphorylation of MEK, ERK1/2, PI3K and AKT in MDA-MB-453 and MDA-MB-231 cells, aligning with previous findings on AG’s anti-HIV effects (Kim et al., 2011). Additionally, AG induced apoptosis in MDA-MB-453 and MDA-MB-231 cells, the elevated Bax/Bcl-2 ratio and cleaved caspase-3 and caspase-9 levels indicated that AG-induced apoptosis is mediated through a caspase-dependent mitochondrial pathway. Therefore, the mechanism shows that AG binds to SRC and inhibits the downstream PI3K-AKT and MEK/ERK signaling pathways, thereby triggering a cascade involving Bax, Bcl-2, caspase-3, and caspase-9 to induce the TNBC cell apoptosis. These results suggest that suppressing SRC-mediated bioactivity is a promising strategy for TNBC therapy.

However, the current study has several limitations. To begin with, the primary pathways that explain the role of AG in TNBC have yet to be thoroughly confirmed and need additional exploration, such as through rescue experiments. Furthermore, *in vivo* research is essential to substantiate these results and assess the viability of AG in the clinical treatment of TNBC. These aspects will be further investigated in future studies.

## Conclusion

In summary, our integrated network pharmacology approach systematically elucidated the molecular mechanisms underlying AG’s anti-TNBC activity. Mechanistic investigations revealed that AG specifically targets SRC kinase, thereby dually suppressing both PI3K/AKT and MAPK/ERK signaling cascades. This coordinated pathway inhibition mediated significant anti-tumor effects through two complementary mechanisms: (1) arresting proliferation via cell cycle blockade and (2) triggering mitochondrial-dependent apoptosis. Structural analysis of the stabilized AG-SRC complex not only provides a rational chemical framework for structure-based drug optimization but also offers mechanistic insights for developing combination therapies targeting SRC-mediated resistance pathways in clinical TNBC management.

## Data Availability

The raw data supporting the conclusions of this article will be made available by the authors, without undue reservation.
