# Supplementary material for: SRC is a potential target of Arctigenin in treating triple-negative breast cancer: based on machine learning algorithms, molecular modeling and in Vitro test
Source: Front Mol Biosci. 2025 Sep 11;12:1644169. doi: 10.3389/fmolb.2025.1644169 (PMC12460110; doi:10.3389/fmolb.2025.1644169)
Supplement: Supplementary file 2 [file Supplementaryfile1.docx]

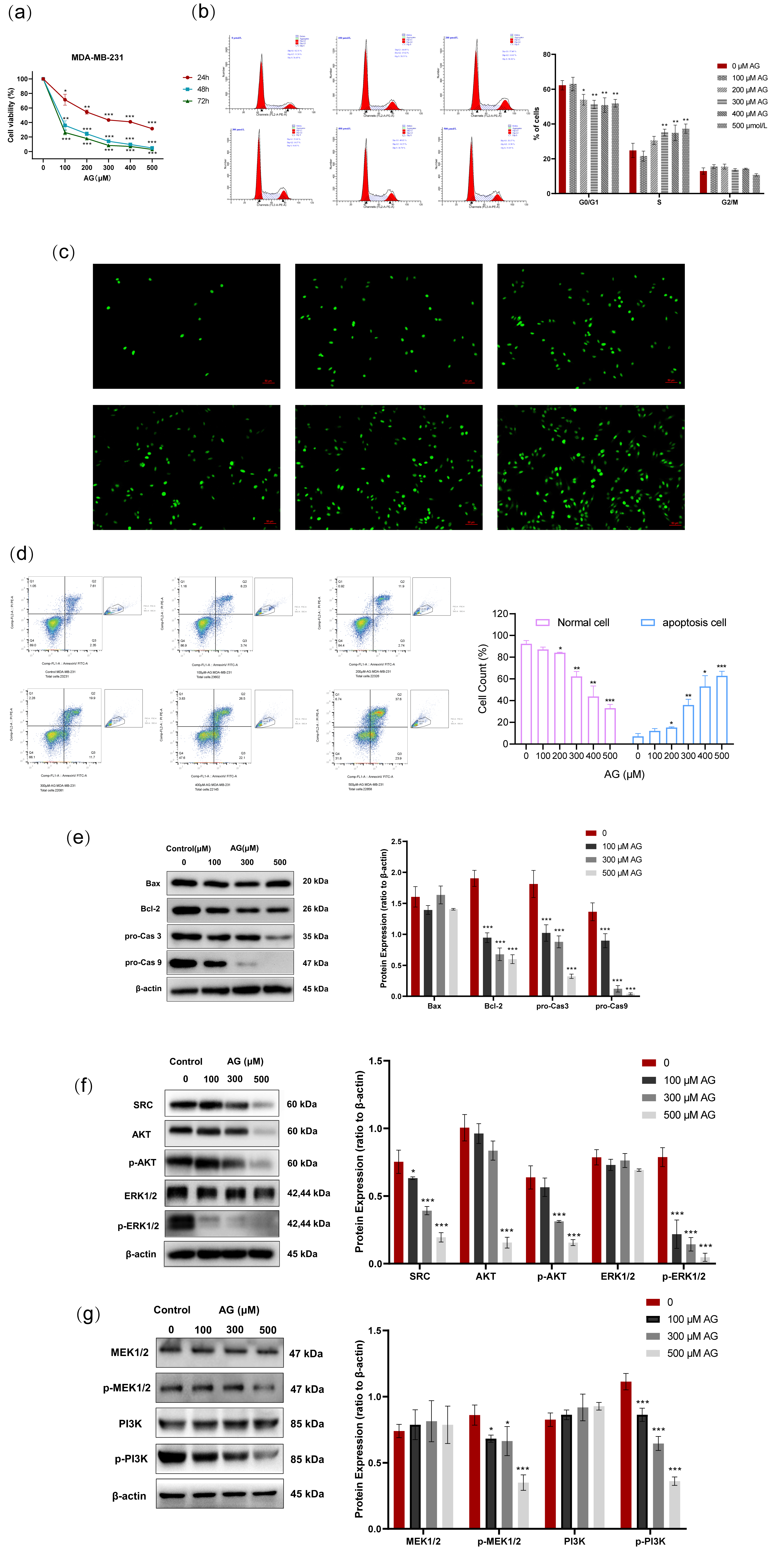


Figure S1. Effects of AG on the MDA-MB-231 cells. a Cell viability was determined by CCK8 assay. b Cell cycle changes were analyzed by FACS based on PI staining. c Cells were incubated with various concentrations of AG for 48 h and stained with fluorescent dye. d Apoptosis rate was analyzed by FACS based on Annexin-FITC/PI staining. d Expression of Bax, Bcl-2, caspase-3, and caspase-9 using western blot. e Impact of AG on the expression of ERK1/2, p-ERK1/2, AKT, p-AKT and SRC. f Impact of AG on the expression of PI3K, p-PI3K, MEK1/2 and p-MEK1/2. 𝑥̅ ± 𝑠, 𝑛 = 3, *, ** and *** indicate 0.01 < P < 0.05, P < 0.01 and P < 0.001 vs. untreated control.
