# Supplementary material for: SRC is a potential target of Arctigenin in treating triple-negative breast cancer: based on machine learning algorithms, molecular modeling and in Vitro test
Source: Front Mol Biosci. 2025 Sep 11;12:1644169. doi: 10.3389/fmolb.2025.1644169 (PMC12460110; doi:10.3389/fmolb.2025.1644169)
Supplement: Supplementary file 3 [file Table3.docx]

TableS3 Validation of Docking Protocol Using Redocking RMSD Values of Known SRC Inhibitors

| PDB ID | RMSD of redocking (Å) |
| --- | --- |
| 1Y57 | 1.94 |
| 2H8H | 1.08 |
| 3EL8 | 0.80 |
| 4MXO | 2.50 |
